# Chronic Losartan Administration Reduces Mortality and Preserves Cardiac but Not Skeletal Muscle Function in Dystrophic Mice

**DOI:** 10.1371/journal.pone.0020856

**Published:** 2011-06-22

**Authors:** Lawrence T. Bish, Mark Yarchoan, Meg M. Sleeper, Jeffrey A. Gazzara, Kevin J. Morine, Pedro Acosta, Elisabeth R. Barton, H. Lee Sweeney

**Affiliations:** 1 Department of Physiology, University of Pennsylvania School of Medicine, Philadelphia, Pennsylvania, United States of America; 2 Division of Cardiology, Department of Clinical Studies, Veterinary Hospital of the University of Pennsylvania, Philadelphia, Pennsylvania, United States of America; 3 Department of Anatomy and Cell Biology, School of Dental Medicine and Pennsylvania Muscle Institute, University of Pennsylvania, Philadelphia, Pennsylvania, United States of America; Istituto Dermopatico de ll'Immacolata, Italy

## Abstract

Duchenne muscular dystrophy (DMD) is a degenerative disorder affecting skeletal and cardiac muscle for which there is no effective therapy. Angiotension receptor blockade (ARB) has excellent therapeutic potential in DMD based on recent data demonstrating attenuation of skeletal muscle disease progression during 6–9 months of therapy in the *mdx* mouse model of DMD. Since cardiac-related death is major cause of mortality in DMD, it is important to evaluate the effect of any novel treatment on the heart. Therefore, we evaluated the long-term impact of ARB on both the skeletal muscle and cardiac phenotype of the *mdx* mouse. *Mdx* mice received either losartan (0.6 g/L) (n = 8) or standard drinking water (n = 9) for two years, after which echocardiography was performed to assess cardiac function. Skeletal muscle weight, morphology, and function were assessed. Fibrosis was evaluated in the diaphragm and heart by Trichrome stain and by determination of tissue hydroxyproline content. By the study endpoint, 88% of treated mice were alive compared to only 44% of untreated (p = 0.05). No difference in skeletal muscle morphology, function, or fibrosis was noted in losartan-treated animals. Cardiac function was significantly preserved with losartan treatment, with a trend towards reduction in cardiac fibrosis. We saw no impact on the skeletal muscle disease progression, suggesting that other pathways that trigger fibrosis dominate over angiotensin II in skeletal muscle long term, unlike the situation in the heart. Our study suggests that ARB may be an important prophylactic treatment for DMD-associated cardiomyopathy, but will not impact skeletal muscle disease.

## Introduction

Duchenne muscular dystrophy (DMD) is a degenerative disorder affecting skeletal and cardiac muscle for which there is no effective therapy [1]. Boys typically present with symptoms of muscle weakness by age five, become wheelchair-bound by early to mid teens, and die from respiratory failure or cardiomyopathy in their late teens to early twenties [2]. One approach to the treatment of DMD involves modulating muscle repair pathways to compensate for the rapid pace of muscle turnover [3]. The inability of muscle regeneration to keep pace with destruction in DMD leads to fibrosis, a process that is mediated largely by transforming growth factor beta (TGF-β) [4], [5]. Increased TGF-β signaling has been documented in both the *mdx* mouse and in the Golden Retriever models of DMD [5], [6].

A recent study demonstrated that antagonism of TGF-β with losartan, an angiotensin II receptor blocker that is known to significantly reduce TGF-β activity in a number of disease models [7], [8], for 6–9 months beginning at 6 weeks of age results in reduced fibrosis in the diaphragm and gastrocnemius muscles and increased forelimb and hindlimb grip strength compared to untreated *mdx* mice. Since losartan is a widely used antihypertensive drug that is known to be safe in humans, this research has generated interest in using losartan as a treatment for patients with DMD [9], [10]. However, cardiac function and fibrosis was not assessed in this study. Therefore, to investigate further the therapeutic potential of losartan in DMD, a disease characterized by both skeletal muscle and cardiac dysfunction, we sought to expand on this previous study by evaluating the functional impact of losartan therapy on both skeletal and cardiac muscle of *mdx* mice after two years of treatment.

## Methods

### Ethics Statement and Animal Use Protocol

All mice were handled in compliance with the *Guide for the Care and Use of Laboratory Animals* published by the National Institutes of Health (NIH publication No. 85–23, revised 1996). All animal studies were approved by the Institutional Animal Care and Use Committee (IACUC) of the University of Pennsylvania (#802238). Male *mdx* mice were weaned at four weeks and randomized to receive standard drinking water (n = 9) or water supplemented with losartan (0.6 g/L) (n = 8) [5]. Water was available ad libitum, and treatment was continued for two years.

### Transthoracic Echocardiography

M-mode echocardiography was performed on mice two years following the inception of losartan treatment under ketamine/xylazine anesthesia using a 15-MHz phased-array probe connected to a Sonos 7500 echocardiographic machine (Philips Medical Imaging, Andover, Massachusetts). In brief, an M-mode cursor was positioned in the parasternal short-axis view perpendicular to the interventricular septum and posterior wall of the LV at the level of the papillary muscles, and M-mode images were obtained for measurement of LV end-diastolic and end-systolic dimension (LVDd and LVDs). The percentage of fractional shortening (%FS) was calculated from the equation%FS  =  [(LVDd – LVDs)/LVDd] ×100. The end diastolic and end systolic volumes, ejection fraction, cardiac output and stroke volume were calculated using the Teicholtz formulas [11]. The same sonographer performed all the studies and resulting calculations and was blinded to the treatment groups of the mice.

### Muscle Function Analysis

The contractile properties of the soleus, EDL, and diaphragm were measured. Mice were anesthetized with ketamine/xylazine (80/10 mg/kg) and exsanguinated. Blood samples were collected, allowed to clot for 2 hours at room temperature, then centrifuged at 2000 x g for 20 minutes to isolate serum. Serum was stored at −80°C, and creatine kinase was measured later using the assay manufactured by Genzyme (Charlottetown, Canada). The EDL, soleus, and diaphragm were then removed, bathed in an oxygenated Ringers solution gas-equilibrated with 95% O_2_, 5% CO_2_, and subjected to isolated mechanical measurements using a previously described apparatus (Aurora Scientific, Ontario, Canada) [12]. After determining optimum length (Lo) by supramaximal twitch stimulation, maximum isometric tetanus was measured.

### Histology

Upon completion of muscle function analysis, muscles were weighed, then rapidly frozen in OCT compound (Tissue Tek, Torrance, CA) in melting isopentane for morphological analysis. Ten micron thick sections were cut, and the resulting slides were stained using standard hematoxylin and eosin (H&E) and Trichrome stains (Sigma, St. Louis, MO).

### Hydroxyproline Analysis

Cardiac and diaphragm fibrosis was assessed by quantifying tissue hydroxyproline content [13]. Tissue amino acid analysis was performed by AAA Laboratories (Mercer Island, WA) using a Beckman 7300 Amino Acid Analyzer coupled with System Gold software following hydrolysis in 6N-HCl/0.05% Mercaptoethanol/0.02% phenol for 20 hours at 115°C.

### Western Blot

Samples obtained for Western blotting were snap-frozen in liquid nitrogen. Specimens were pulverized, homogenized in 10 volumes of triple-detergent lysis buffer [50 mM Tris, pH 8.0, 0.1% SDS, 1.0% Triton X-100, 0.5% DOC, 5 mM EDTA, 50 mM DTT, 0.4 tablet/10 mL Complete Protease Inhibitor Cocktail Tablets (Roche, Indianapolis, IN)], and centrifuged at 13,000 rpm for 5 minutes. Protein concentration of the supernatant was then determined using the BioRad Protein Assay (Hercules, CA), and a 50 ug aliquot of each sample was denatured using an equal volume of 2X sample loading buffer (130 mM Tris, pH = 8.0, 20% glycerol, 4.6% SDS, 2% DTT, 0.02% bromophenol blue) at 100°C (5 min) then fractionated electrophoretically on a 4–20% SDS-polyacrylamide gel (Lonza, Rockland, ME). Proteins were then electroblotted onto a polyvinylidene fluoride membrane (Immobilon-P, Millipore, Bedford, MA) using the iBlot transfer apparatus (Invitrogen, Carlsbad, CA). The membrane was subsequently blocked by incubating in Tris-buffered saline containing 5% nonfat dry milk and 0.05% Tween 20. Immunoblotting was performed to detect Nav1.5 (1∶100, Abcam, Cambridge, MA); connexin 40 (1∶100, Abcam, Cambridge, MA); and GAPDH (1∶2000, Santa Cruz Biotechnology, Santa Cruz, CA). Detection was performed using the Super Signal West Pico Chemiluminescent Substrate kit (Pierce, Rockford, IL).

### Statistics

Mean values from each experimental group were compared using the two-tailed Student's t test or one-way ANOVA with Student-Neuman-Keuls post hoc analysis as appropriate. The Kaplan-Meier survival curve was generated using Graph-Pad Prism software with log-rank analysis (La Jolla, CA).

## Results

### Study Design and Mortality

**Figure 1:**
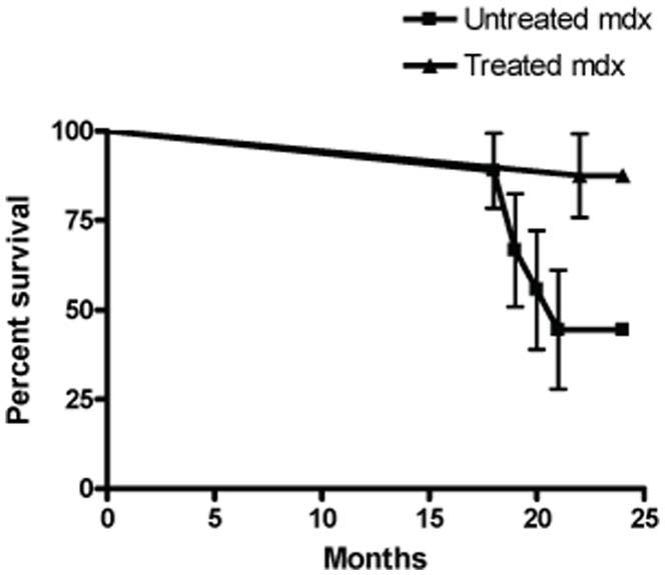
Survival in treated and untreated *mdx* mice at two years. The survival curves for treated and untreated *mdx* mice are significantly different(p = 0.05). 88% of treated mice were alive at two years compared to only 44% of untreated mice. Data represent mean±SEM.

Male *mdx* mice were weaned at four weeks and randomized to receive either standard drinking water (n = 9) or drinking water supplemented with losartan (0.6 g/L) (n = 8) for two years [5]. Water was provided ad libitum. By the study endpoint, 88% (7 out of 8) of treated mice were alive compared to only 44% (4 out of 9) of untreated mice (**Figure 1**) (p = 0.05).

### Skeletal Muscle Analysis

**Figure 2:**
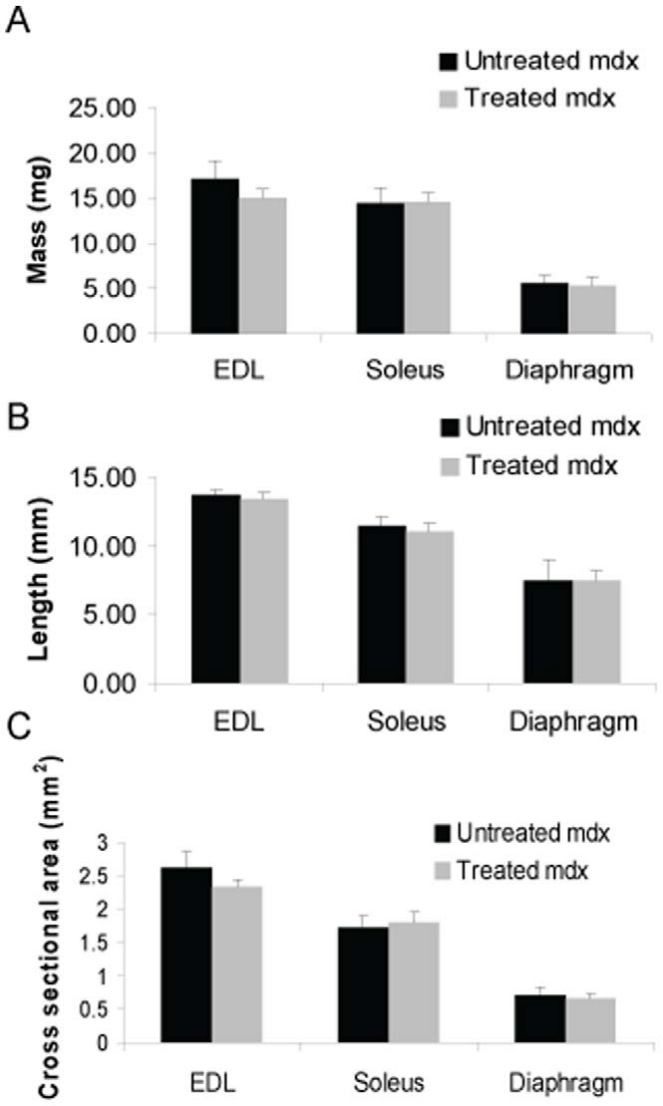
Skeletal muscle morphology in *mdx* mice at two years. (a) Mass, (b) length, and (c) cross sectional area of the extensor digitorum longus (EDL), soleus, and diaphragm were quantified at two years in treated and untreated *mdx* mice . No significant differences were noted. Data represent mean±SD.

**Figure 3:**
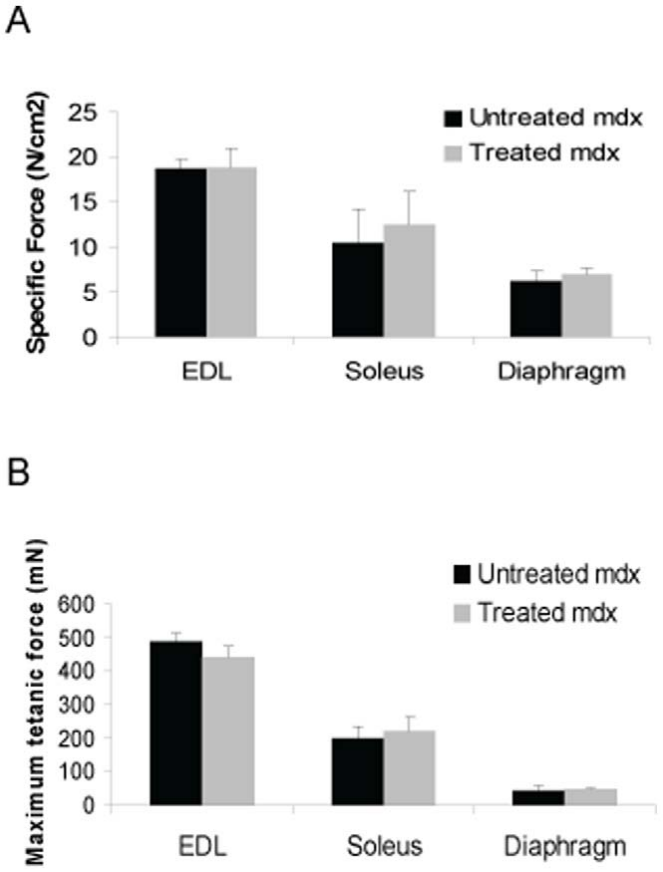
Skeletal muscle function in *mdx* mice at two years. (a) Specific force and (b) maximum tetanic force of the extensor digitorum longus (EDL), soleus, and diaphragm were quantified at two years in treated and untreated *mdx* mice. No significant differences were noted. Data represent mean±SD.

The extensor digitorum longus (EDL), soleus, and diaphragm were analyzed at 2 years. There was no significant difference in mass, length, or cross sectional area in losartan-treated mice vs. untreated (**Figure 2**). Furthermore, specific force and maximum tetanic force measurements of the EDL, soleus, and diaphragm muscles were not significantly different in treated vs. untreated animals (**Figure 3**).

**Figure 4:**
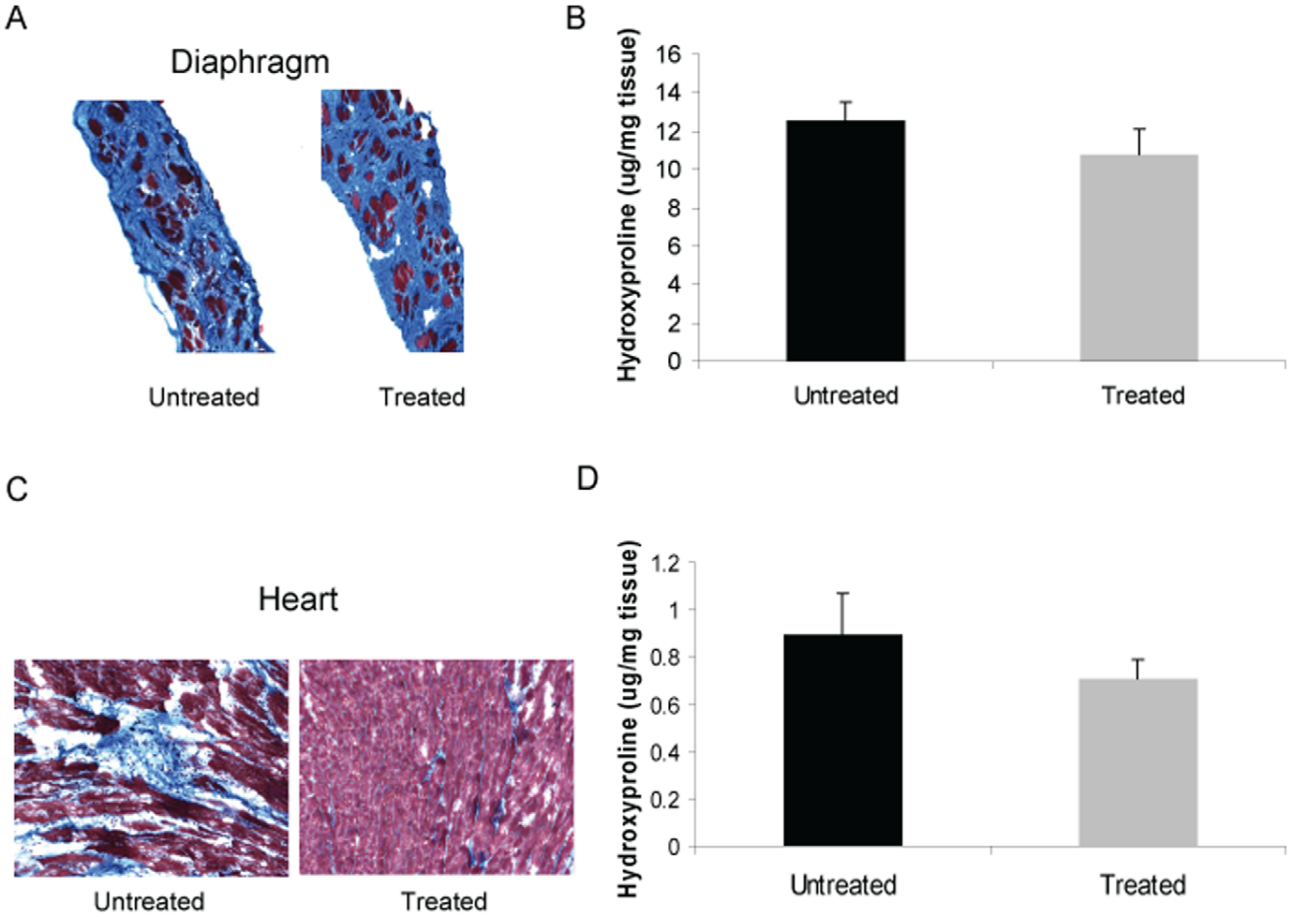
Fibrosis in *mdx* mice at two years. Trichrome stain was performed and tissue hydroxyproline content was determined to measure fibrosis in *mdx* mice at two years. (a) Trichrome stain and (b) tissue hydroxyproline content of the diaphragm, as well as (c) Trichrome stain and (d) tissue hydroxyproline content of the heart are demonstrated. Note that there was a trend towards reduced hydroxyproline tissue content in the heart with losartan treatment (p = 0.08). Data represent mean±SD.

Hematoxylin and eosin (H&E) and Trichrome staining was performed on the EDL, soleus, quadriceps, and diaphragm. No significant differences were noted in terms of the number of centrally located nuclei (H&E) (data not shown) or extent of fibrosis (Trichrome) (**Figure 4A**). Fibrosis was also quantified by measuring the hydroxyproline content of the diaphragm. Losartan treatment did not reduce fibrosis as measured by hydroxyproline content (**Figure 4B**).

Serum creatine kinase (CK) was measured in losartan-treated and untreated mice, and no significant difference was noted between the two groups (data not shown).

### Cardiac Muscle Analysis

Cardiac function was assessed via echocardiography. Untreated *mdx* mice exhibited left ventricular dilation (i.e, increased left ventricular inner diameter in diastole and end diastolic volume) and systolic dysfunction (i.e., decreased ejection fraction and fractional shortening) at two years, while *mdx* mice treated with losartan exhibited preserved cardiac geometry with normal systolic function (**Table 1**).

**Table 1:** Cardiac Function As Assessed By 2-D Echocardiography At 2 Years.

	HR^a^(bpm)	IVS^b^(cm)	LVIDd^c^(cm)	LVFW^d^(cm)	LVIDs^e^(cm)	FS^f^(%)	EDV^g^(ml)	ESV^h^(ml)	EF^i^(%)	LVmass^j^(g)	CO^k^(L/min)	SV^l^(ml)
Con^m^	334±149^n^	0.09±0.00	0.40±0.01	0.10±0.01	0.33±0.03	17±7.9	0.16±0.01	0.10±0.03	40±17	0.72±0.02	0.02±0.01	0.06±0.03
Los^o^	282±103	0.11±0.01	0.35±0.04	0.11±0.02	0.21±0.03	40±7.0	0.11±0.04	0.03±0.01	74±10	0.71±0.02	0.02±0.02	0.07±0.05
P^p^	0.5026	**0.0390**	**0.0262**	0.3556	**0.0001**	**0.0007**	**0.0388**	**0.0004**	**0.0021**	0.4798	0.4910	0.6873

a HR: heart rate;^ b^IVS: interventricular septum;^ c^LVIDd: left ventricular inner diameter in diastole;^ d^LVFW: left ventricular free wall;^ e^LVIDs: left ventricular inner diameter in systole;^ f^FS: fractional shortening; ^g^EDV: end diastolic volume; ^h^ESV: end systolic volume; ^i^EF: ejection fraction; ^j^LV: left ventricular; ^k^CO: cardiac output; ^l^SV: stroke volume;^ m^Con: control treatment; ^n^Values reported as mean ± standard deviation;^ o^Los: losartan treatment; ^p^p: p value.

Cardiac fibrosis was assessed by Trichrome stain (**Figure 4C**) and quantified by measuring tissue hydroxyproline content. There was a trend towards reduction of fibrosis with losartan treatment (p = 0.08) (**Figure 4D**).

**Figure 5:**
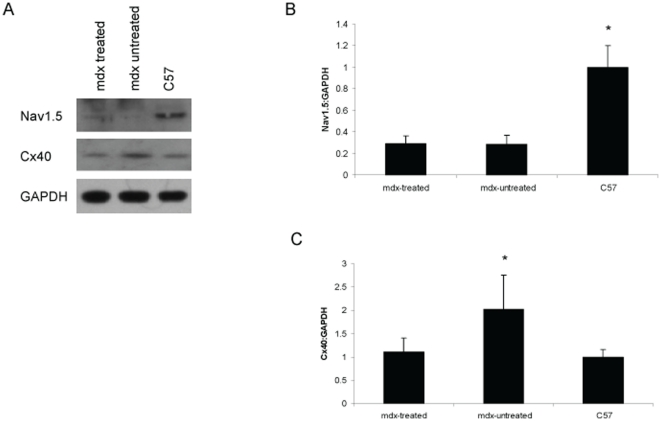
Expression of conduction-related proteins in the heart at two years. (a) Western blot was performed to quantify levels of the sodium channel Nav1.5 and gap junction component connexin 40 (Cx40) in the hearts of treated *mdx*, untreated *mdx*, and healthy C57 mice. (b) Nav1.5 expression was significantly decreased in untreated *mdx* mice compared to healthy C57 controls, and losartan treatment did not restore Nav1.5 expression. *p<0.05 vs. treated *mdx* and untreated *mdx*. (c) Cx40 expression was significantly increased in untreated *mdx* mice compared to healthy C57 controls, and losartan treatment normalized expression. *p<0.05 vs. treated *mdx* and C57 control. Data represent mean±SD.

Expression of proteins involved in electrical conduction in the heart was analyzed by Western blot **(** **Figure 5A** **)**. Protein levels of the sodium channel Nav1.5 were significantly decreased in untreated *mdx* hearts compared to healthy C57 mouse hearts; losartan treatment did not restore Nav1.5 expression in *mdx* mice (**Figure 5B**). Expression of the gap junction protein connexin 40 (Cx40) was increased in untreated *mdx* hearts compared to healthy C57 mouse hearts; losartan treatment resulted in normalization of Cx40 expression (**Figure 5C**).

## Discussion

DMD is an inherited disorder characterized by skeletal muscle and cardiac pathology for which there is no effective treatment. Angiotensin receptor blockade (ARB) via losartan has been proposed to have therapeutic potential for DMD based on recent data demonstrating attenuation of skeletal muscle disease progression during 6–9 months of therapy in the *mdx* mouse [5]. However, cardiac disease was not evaluated in that study. Since cardiac-related death is major cause of mortality in DMD [2], it is important to evaluate the effect of any treatment directed at skeletal muscle on the heart. In this study, we evaluated the long-term impact of ARB on both the skeletal muscle and cardiac phenotype of the *mdx* mouse model of DMD following two years of losartan treatment. We report that two years of ARB reduced mortality and preserved cardiac function in *mdx* mice. However, we were unable to detect any preservation of function or reduction of fibrosis in these aged *mdx* skeletal muscles at two years, contrary to what was reported in younger *mdx* mice [5].

Several recent studies have described the phenotype of aged *mdx* mice. Van Erp *et al* followed *mdx* mice from 3–18 months and characterized the development of cardiomyopathy during this period [14]. They found that although cardiac collagen content and macrophage infiltration was significantly increased by 6 months, basal ventricular function was not significantly decreased until 18 months. They concluded that this decreased cardiac function occurs secondary to ongoing cell death, inflammation, fibrosis, and eventual cardiac remodeling, and they recommend that studies aimed at evaluating cardiac function in *mdx* mice should be performed in animals greater than 18 months old. A second group followed *mdx* mice for 20–22 months and evaluated the cardiac phenotype in untreated *mdx* mice and in *mdx* mice carrying a mini-dystrophin transgene [15]. They found that aged *mdx* mice exhibit cardiac fibrosis, ECG abnormalities, systolic dysfunction, and decreased exercise performance, and they report that mini-dystrophin expression can partially improve this phenotype.

Other studies have focused on analysis of the skeletal muscle phenotype of aged *mdx* mice. Mouisel *et al* followed *mdx* mice for 18–24 months and found that the skeletal muscles of these old mice were characterized by decreased absolute force, atrophy, and decreased regenerative capacity compared to 5 month old *mdx* mice [16]. Another group used adeno-associated virus to deliver micro-dystrophin systemically to 20 month old *mdx* mice and reported that this approach was effective not only in halting disease progression but also in improving muscle structure and function by 24 months, thus demonstrating that it is possible to improve the phenotype of aged *mdx* mice [17]. The investigators also suggested that the aged *mdx* mouse is the small animal model that most closely mimics the severe phenotype of Duchenne muscular dystrophy (DMD) and therefore is a superior model to the young *mdx* mouse when evaluating novel therapeutic strategies for DMD.

In this study, our goal was to evaluate the effects of angiotensin receptor blockade (ARB) on both the cardiac and skeletal muscle phenotype of the aged *mdx* mouse, and we chose 24 months as an endpoint. This endpoint seemed ideal based on the existing literature since it would allow us not only to evaluate cardiac function at a timepoint after which basal ventricular dysfunction had been previously reported [14] but also to evaluate the skeletal muscle phenotype in its most severe state [17]. Our data suggest that while ARB may be an effective long-term therapy for DMD-associated cardiomyopathy, it will not be effective as a long-term treatment for the skeletal muscle disease.

It is unclear why no long term benefit of losartan was observed in skeletal muscle. It may be that fibrosis early in the disease process is driven largely by angiotensin II, but that over time, other pathways become dominant in skeletal muscle. This would allow the rate of fibrosis to increase despite ARB at a time when dystrophic satellite cells are experiencing a decline in regenerative capacity [16]. Future investigation is needed identify these pathways and to determine the conditions under which losartan is efficacious in skeletal muscle.

Although we did not observe any benefit of losartan in skeletal muscle function, cardiac function and geometry was preserved, and mortality was decreased after two years of angiotensin receptor blockade. This result is in agreement with a recent study that demonstrated improved cardiac function, but not improved skeletal muscle function, following 6 months of losartan treatment in *mdx* mice [18]. Our data extend the findings of this previous study and suggest that angiotensin II signaling is a major contributor to cardiac disease progression throughout the entire disease course. The mechanism underlying this cardiac benefit may be related to a reduction in fibrosis since we observed an approximately 20% reduction in cardiac fibrosis with losartan treatment that trended towards significance (p = 0.08). This is in agreement with the losartan-mediated reduction in cardiac fibrosis via TGF-β anatagonism that has been reported in several other models of cardiomyopathy [18], [19], [20], [21], [22]. The reduction in fibrosis may not have reached significance in our study due to (i) relatively small study size since five out of nine untreated mice had died by the two year endpoint, (ii) increased cumulative cardiac fibrosis in our mice that were 6–12 months older than the mice studied in previous reports, or (iii) a combination of the two.

Cardiac function may also have been improved by a mechanism independent from a reduction in fibrosis. Previous studies have reported that the sodium channel Nav1.5 is decreased in the *mdx* heart [23] and that the gap junction component Cx40 is increased in both the *mdx* heart [23] and in the hearts of dilated cardiomyopathy patients [24]. These alterations likely contribute to the abnormal ECG findings and arrhythmias previously described in *mdx* mice [23]. In agreement with these previous reports, we found that Nav1.5 is decreased and Cx40 increased in the hearts of *mdx* mice compared to healthy C57 mice. We also found that while ARB did not alter expression of Nav1.5, it did restore Cx40 expression to levels found in the healthy C57 heart. Therefore, normalization of the function of gap junctions may contribute to the observed improvement in cardiac function and reduced mortality in *mdx* mice treated with ARB. Future investigation in needed to determine the effect of ARB on arrythmogenesis and on the abnormal ECG findings in *mdx* mice [23].

It has also been reported that defects in calcium handling secondary to dysregulation of the sarcoplasmic reticulum calcium ATPase 2a (SERCA2a) and phospholamban (PLB) are present in the failing hearts of humans and animals [25], [26]. Results from a recent study suggest that ARB in the heart can lead to increased expression and activity of SERCA2a via modulation of protein kinase A and Ca^2+^/calmodulin-dependent protein kinase II signaling [22]. Further investigation is needed to determine if this mechanism contributes to improved cardiac function in *mdx* mice treated with losartan.

In summary, we report that two years of ARB in the *mdx* mouse preserves cardiac function and decreases mortality. No benefit in terms of skeletal muscle morphology or function was observed, suggesting that pathway(s) other than angiotensin II are dominant in driving skeletal muscle fibrosis in late stage disease. In addition, our study suggests that ARB has potential as a treatment for DMD-associated cardiomyopathy. Future investigation should be directed at evaluating losartan in large animal models and patients with DMD, as well as comparing effectiveness of ARB and angiotension converting enzyme (ACE) ACE inhibition.
